# Feasibility analysis of high pitch cervical spine CT in uncooperative patients with acute cervical spine trauma: An initial experience

**DOI:** 10.1097/MD.0000000000030785

**Published:** 2022-09-30

**Authors:** Juntao Cao, Na Xie, Pingkang Qian, Ming Hu, Jianchun Tu

**Affiliations:** a Department of Radiology, Kunshan Hospital of Traditional Chinese Medicine, Jiangsu Province, China; b Department of Medical Imaging, Kunshan Maternal and Child Health Hospital, China; c Trauma Center, Kunshan Hospital of Traditional Chinese Medicine, China.

**Keywords:** cervical CT, cervical spine trauma, high pitch, uncooperative

## Abstract

Cervical computed tomography (CT) often suffers from examination failure in uncooperative patients with acute cervical spinal trauma. Therefore, this study aimed to evaluate the feasibility of using high-pitch cervical CT (HP-CT) in such populations. A total of 95 patients with acute neck/head-neck trauma who underwent HP-CT (n = 29) or standard cervical CT (SD-CT, n = 66) from October 2020 to June 2021 were included in this study. Differences in patient characteristics between the HP-CT group and the SD-CT group were firstly compared. Then, the objective image quality based on the mean score of the signal-to-noise ratio (SNR)/contrast noise ratio (CNR) was evaluated, while double-blind five-point scoring was adopted for the subjective evaluation. Finally, radiation doses in HP-CT and SD-CT were compared. Furthermore, the Student *t* test and/or Mann–Whitney *U* test were performed to analyze differences in patient characteristics, image quality, and radiation dose between the two regimes. A total of 17 cases of cervical spine fractures were found in 95 patients, including 6 cases in the HP-CT group and 11 cases in the SD-CT group. The average age of patients who received HP-CT was higher than that of those who received SD-CT, and the scan time using HP-CT was shorter than that SD-CT. The differences were statistically significant (both, *P* < .05). In addition, there was no significant difference between HP-CT and SD-CT in terms of sex, body mass index, field of view (FOV), and scan length (all *P* > .05). The SNR/CNR at the middle and upper neck was not significantly different between HP-CT and SD-CT (all *P* > .05). However, the SNR/CNR at the lower neck in HP-CT was lower than that in SD-CT (all *P* < .05). There was no significant difference in the subjective scores between HP-CT and SD-CT images in both the soft tissue and bone window (*P* = .129 and 0.649, respectively). The radiation dose in HP-CT was lower than that in SD-CT (all *P* < .05). With a scan time reduction of 73%, radiation dose reduction of 10%, and similar image quality, high-pitch cervical CT was of feasibility to evaluate cervical spine injury in uncooperative patients with acute cervical spine trauma.

## 1. Introduction

Cervical spine injury (CSI) accounts for approximately 2% to 15% of the whole-body trauma, of which 10% to 20% cases would have spinal cord injury.^[1-3]^ It may lead to catastrophic consequences if the CSI was not found immediately. Therefore, it is crucial to quickly and accurately identify CSI in patients with head or neck trauma. National Emergency X-radiography utilization study (NEXUS) and the Canadian C-Spine Rules (CCR) are generally followed in the preliminary assessment of CSI for patients with cervical spine trauma since they have important guiding significance in excluding unnecessary imaging examinations in patients with low-risk CSI ^[4]^. However, the application of NEXUS and CCR requires the patient to be conscious and alert. Furthermore, there is no consensus on the evaluation of uncooperative patients with cervical spine trauma.^[5]^ In addition, the physical examination for this situation is not necessarily reliable.^[6, 7]^ The risk of CSI is higher in patients who cannot be evaluated than that in those who are sober.^[2]^

A large number of studies have demonstrated that cervical computed tomography (CT) is one of the most effective approaches to the clearance of CSI, especially after the appearance of spiral CT.^[8-11]^ However, the limitation of standard cervical CT (SD-CT, provided by the vendor) occurs when it is applied to uncooperative patients with acute cervical spine trauma.^[5]^ Although the cervical collar is usually used to immobilize an injured cervical spine, some unpredictable movements (such as groaning or trembling to result from trauma, unconsciousness/excitement caused by alcohol or drugs, and mental disorders) would occur in patients who need further evaluation by CT, thus posing challenges to SD-CT examination. Hence, a repeated cervical CT scan has to be performed in some cases, which is not uncommon in practice.^[10]^ The pitch in CT was defined as the distance the table-bed moved per tube rotation divided by the collimation width. High-pitch mode CT can significantly reduce the movement artifact by reducing the scanning time, and providing a high-quality image.^[12]^ To the best of our knowledge, there are few studies on the application of high-pitch CT applied to the cervical spine. In this study, it was assumed that the clearance of CSI in uncooperative patients with acute cervical spine trauma can be determined by high-pitch cervical CT (HP-CT).

The purpose of the present study was to evaluate the feasibility concerning exam efficiency, image quality, and radiation dose of HP-CT in uncooperative patients with acute cervical spine trauma by comparing it with the SD-CT received by alert patients.

## 2. Materials and Methods

### 2.1. Study population

The patients transferred to the trauma center of our institution due to acute neck or head-neck trauma from January 2020 to June 2021 were pre-registered. Besides, a preliminary assessment of CSI was performed on all subjects by the emergency physicians based on NEXUS, CCR, or experience. Except for patients with low risk of CSI, all other patients would receive cervical CT examination for further evaluation according to the following criteria: patients in an unconscious or hyperactive state caused by trauma, alcohol, or drugs; patients who were conscious, but showed involuntary movement, such as groaning or shaking; patients with mental disorders or related medical history; patients who failed in the first scan in SD-CT, and need a repeated cervical CT. In brief, patients who met any one or more of (1) to (4) received HP-CT. Otherwise, SD-CT was adopted.

The present study was reviewed by the medical ethics committee of our institution (No. KZY2019-50), and written informed consent was obtained from all subjects.

### 2.2. CT scan protocol and data process

The cervical CT of all subjects was performed on a 128-slice spiral CT (Somatom Definition AS+; Siemens Healthcare, Forchheim, Germany). Apart from the pitch, protocols for the SD-CT and HP-CT were the same on both CT scanners as follows: tube voltage 120 kV, reference tube current 330 mA with CARE Dose4D, thickness 3.0 mm, spacing 3.0 mm, collimation width 128 × 0.6 mm, and tube rotation time 1.0 second; range: from the skull base to the 1st thoracic vertebra; direction: head-foot; field of view (FOV): 16 to 20 cm. The pitch in SD-CT and HP-CT examination was 0.8 and 1.5, respectively, while the images were reconstructed in the soft-tissue window (I30 s) and the bone window (I70 h) in the axial direction, with a thickness of 1.0 mm and an increment of 1.0 mm, and then transmitted to the post-processing workstation for reconstruction [including multi-planar reconstruction (MPR) and volume rending (VR)]. Then, the CT images (both in the soft-tissue/bone window images and MPR/VR) were sent to the Picture Archiving and Communication Systems (PACS) (PACS 2.0, Neusoft, Shenyang, Liaoning, China). Afterward, the professional display (MDNG 6121, Barco, Kortrijk, Belgium) was used for diagnosis.

### 2.3. Image quality evaluation

1. Objective evaluation

**Figure. 1:**
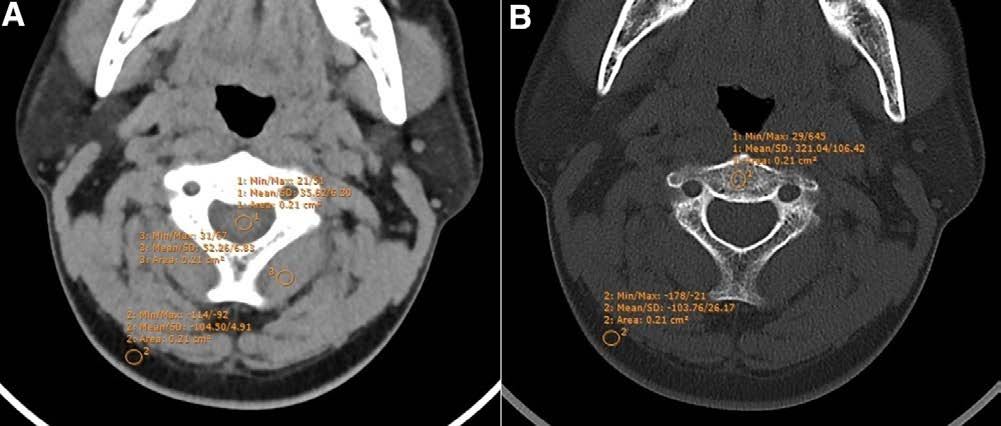
Example of ROIs measurement. The ROIs were placed at the dural sac (A 1), paraspinal muscles (A 3), subcutaneous fat (A 2, B 2), and cervical trabecular bone (B 1) (A: soft tissue window CT image; B: bone window CT image). CT = computed tomography, ROI = regions of interest.


The signal-to-noise ratio (SNR) and contrast noise ratio (CNR) were used for the objective evaluation of image quality. The evaluator will select cervical spine CT images at the upper, middle, and lower neck (corresponding to the C2, C4, and C7 vertebral planes, each of which contains images both in the soft-tissue and bone windows) for image quality evaluation, and the circular regions of interest (ROI: 20–25 mm^2^) placed on the corresponding slices were also adopted. These ROIs were placed in the subcutaneous homogeneous fat, dural sac, paraspinal muscles, and cervical trabecular bone to obtain the corresponding CT value (HU), and the standard deviation of the CT value of the fat on the same plane was taken as the noise value (NOISE). Apart from that, HU/NOISE was measured by the commercial software (Sante DICOM Viewer Pro, Santesoft, Nicosia, Cyprus). Here, it should be noted that each slice was measured twice, and the mean value was employed for statistical analysis. The measurement examples were shown in Figure 1. Based on a previous study,^[14]^ the formulas for SNR and CNR were as follows:

$$\begin{array}{l} SNR_{ROI}=\frac{HU_{ROI}}{SD\;\left(\;fat\right)},\;CNR_{ROI}=\frac{HU_{ROI}-HU_{fat}}{SD\;\left(\;fat\right)} \end{array}$$

The SNR/CNR of the dural sac and paraspinal muscles was obtained from the images in the soft-tissue window, while that of the trabecular bone was acquired from the images in the bone window.

1. Subjective evaluation

The subjective evaluation of image quality was performed by two radiologists who had 7 and 13 years of experience in the diagnosis of musculoskeletal imaging, respectively using the double-blind five-point scoring, and the mean score of two observers was calculated as the subjective score for image quality. As for the scoring criteria, it is shown as follows: 5 = no artifacts, sufficient confidence in the diagnosis; 4 = almost no artifacts, sufficient confidence in the diagnosis; 3 = few artifacts, moderate confidence in the diagnosis; 2 = moderate artifacts, insufficient diagnostic confidence; 1 = severe artifacts, no diagnostic confidence. In addition, scoring criteria training was performed for observers before the final image quality evaluation was made, while the diagnostic report of cervical spine CT was conducted by the radiologists with experience in musculoskeletal imaging after the image quality assessment.

### 2.4. Parameters of CT scan and radiation dose

The CT scan parameters consisting of FOV, scan time, and scan length and the radiation dose parameters composed of mAs, volume CT dose index (CTDIvol), and dose–length product (DLP) were recorded from the scan interface and the scan protocol respectively. Furthermore, the effective dose was obtained by multiplying DLP with the absorption coefficient of 0.0059.^[15]^

### 2.5. Statistical analysis

The statistical analysis was performed via the available software (IBM SPSS Statistics, version 19.0, Chicago, IL). Other than that, the Kolmogorov–Smirnov test was employed for normal distribution, and the data were expressed as mean ± SD when normal distribution was confirmed, or median (Q25, Q75) when not. Besides, the Student *t* test or Mann–Whitney *U* test was conducted for comparing the differences, while LSD and Tamhane T2 were employed for multiple analysis corrections. Moreover, box plots were depicted to describe the SNR/CNR of the ROI at the upper, middle and lower neck, and the *P* value of < .05 was considered statistically significant.

## 3. Results

### 3.1. Overall

**Figure. 2:**
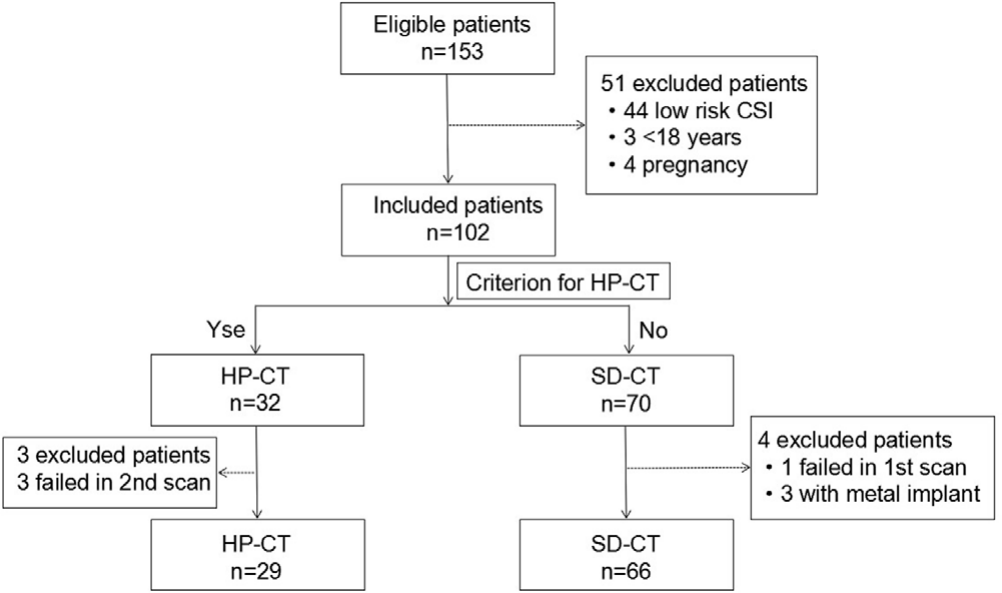
Process of the patients’ enrollment. Note: CSI = cervical spine injury, HP-CT = high-pitch cervical CT, SD-CT = standard cervical CT.

From January 2020 to June 2021, 153 patients with acute cervical spine trauma were transferred to the trauma center of our institution. A total of 51 cases composed of 44 cases of low-risk CSI, 4 cases of pregnant women, and 3 cases of adolescents were excluded from CT examination after initial evaluation by emergency physicians. Hence, 102 patients who needed to undergo cervical CT examination for the clearance of CSI accepted cervical spine CT. Among the 32 cases included for HP-CT, three cases were excluded because of failure in the first two scans for involuntary movement; one patient failed due to unforeseen movement in the first scan, and three with metal implants in the SD-CT group were rejected. Finally, 95 cases were qualified for further analysis. The patient management process was displayed in Figure 2.

**Figure. 3:**
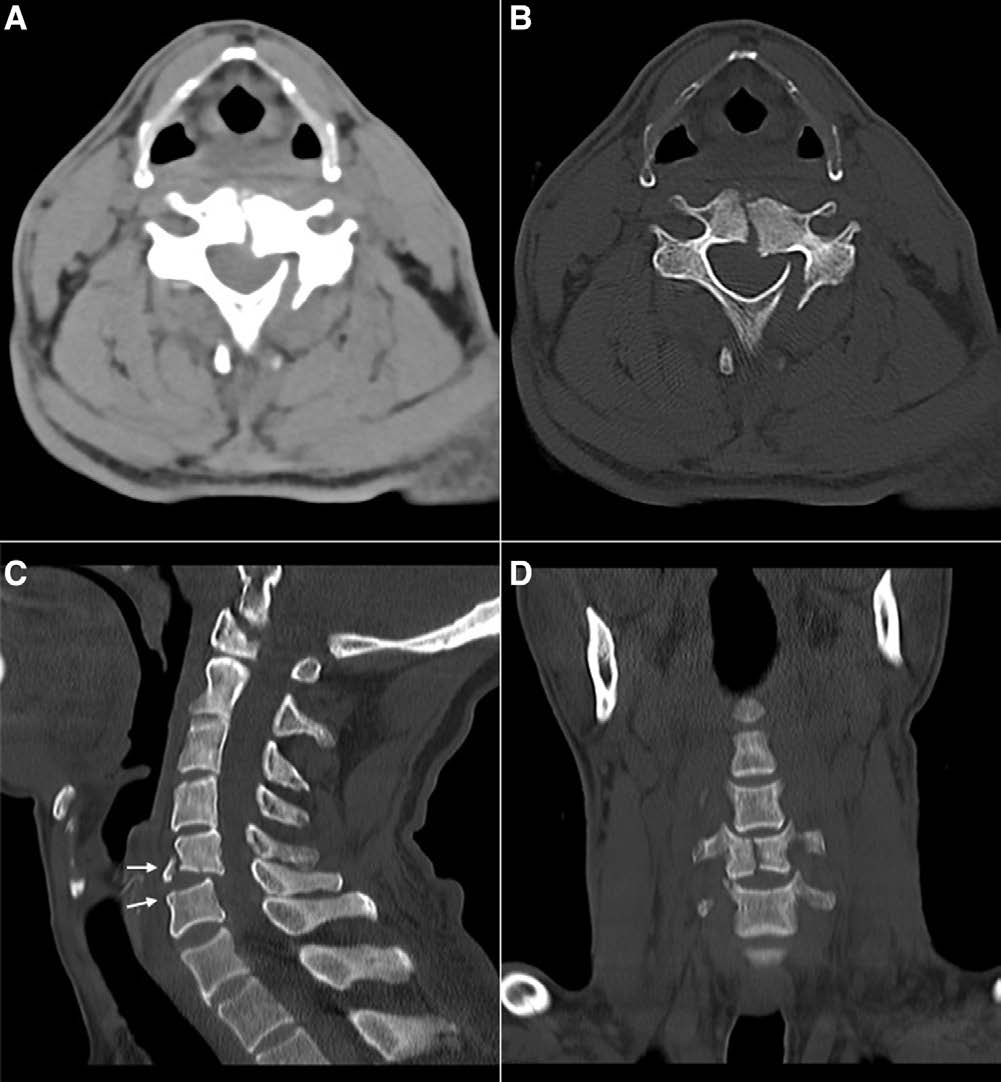
A 65-year-old male intoxicated patient with acute neck trauma received a high pitch cervical CT. The fractures in the C5 vertebral body and its left lamina were shown in A (image in soft tissue window on axial plane, WW450/WL300) and B (image in bone window on axial plane, WW1500/WL450). The dural sac was compressed by the displaced vertebrae， and also the left paravertebral muscle was involved. Some additional details were shown on MPR images, including an avulsion fracture at the lower front of the C5 vertebra (C: sagittal reformatted bone window CT image, WW/WL same as B), a complete fracture in longitudinal of the C5 vertebral body, and a minor fracture at the upper-right edge of the C6 vertebral body (D: coronal reformatted bone window CT image, WW/WL same as B) (white arrow). CT = computed tomography.

**Figure. 4:**
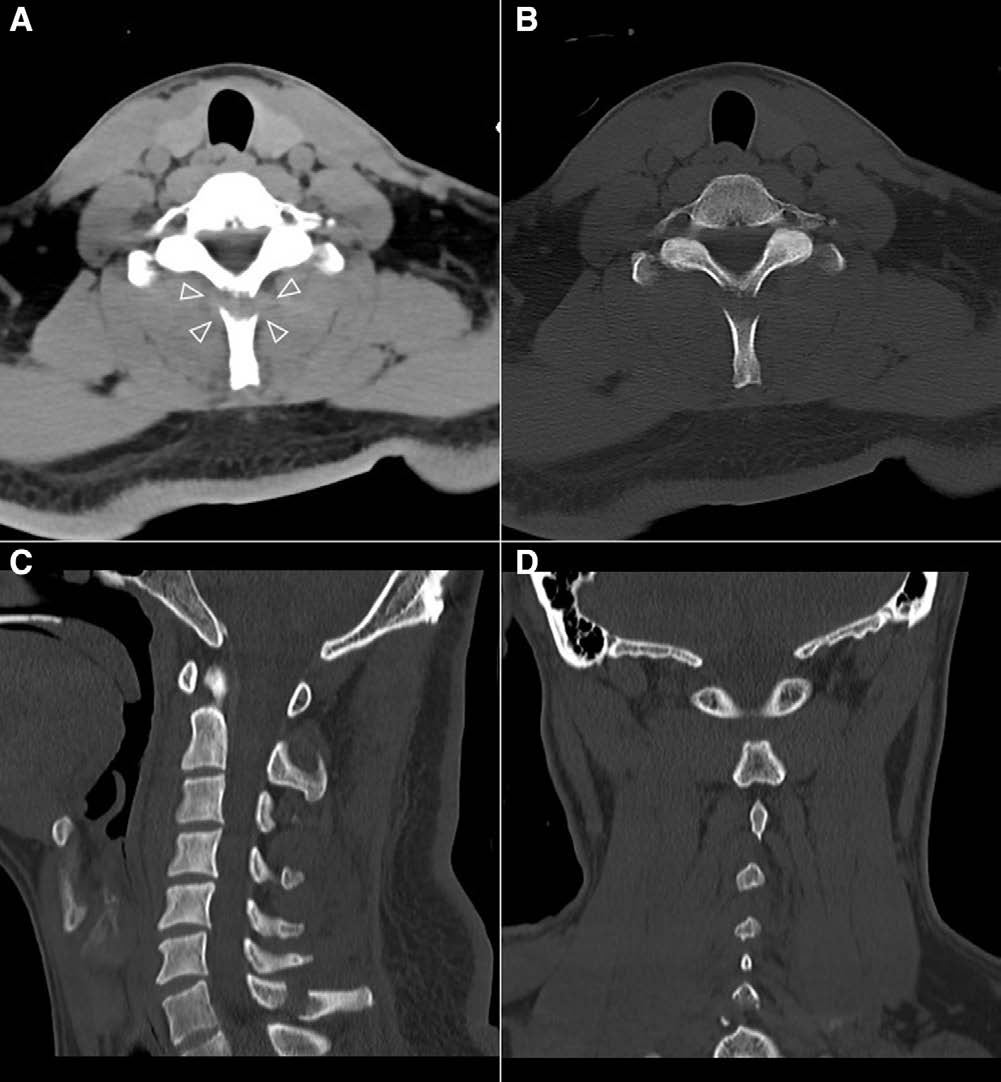
A 35-year-old male alert patient who suffered from a fall injury received a standard cervical CT. The fracture in the C6 spinous process was shown in A (image in soft tissue window on axial plane, WW450/WL300) and B (image in bone window on axial plane, WW1500/WL450). The localized iso-high density shadow at the end of the fracture suggests hematoma formation (△). More detailed spatial anatomical information of the fracture can be better displayed in the MPR(C: sagittal reformatted bone window CT image; D: coronal reformatted bone window CT image, all WW/WL same as B), including the alignment and stability of the fracture. CT = computed tomography.

A total of 95 patients received 100 cervical CT scans, among which 66 scans were performed for 66 cases in SD-CT, and 34 scans were executed for 29 cases in HP-CT. In the HP-CT group, 24 cases succeeded in the first scan, and another 5 cases succeeded in the second scan. Patients who underwent HP-CT were older than those in the SD-CT group. Compared with SD-CT, the scan time of HP-CT was shorter. The differences were statistically significant (both, *P* < .05). However, there was no significant difference in gender distribution, body mass index, FOV, and scan length between HP-CT and SD-CT groups (all, *P* > .05). Besides, the characteristics of patients who underwent HP-CT and SD-CT were presented in Table 1. A total of 17 cases of cervical fractures were found by both HP-CT (6 cases) and SD-CT (11 cases), which included eight cases in the vertebral body, four cases in the spinous process, and five cases in both the vertebral body and the spinous process (Figs. 3 and 4).

**Table 1:** Patient characteristics for SD-CT and HP-CT.

Characteristic	SD-CT (n = 66)	HP-CT (n = 29)	*P* value
Age (yr)	46 ± 14	53 ± 16	.033
Sex male/female	35/31	18/11	.416
BMI	24.68 ± 1.83	24.60 ± 1.49	.837
FOV (cm)	18.29 ± 2.62	18.34 ± 2.01	.082
Scan length (mm)	166.27 ± 17.79	164.28 ± 15.31	.601
Scan time (s)	5.20 ± 0.62	1.39 ± 0.18	<.001

BMI = body mass index, cm = centimeter, FOV = field of view, HP-CT = high pitch cervical CT, mm = millimeter, s = second, SD-CT = standard cervical CT.

### 3.2. Image quality evaluation

**Figure. 5:**
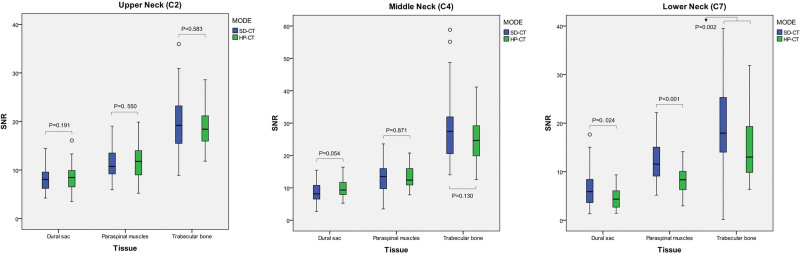
Comparison of SNR of three tissues at the upper, middle, and lower neck (corresponding to the C2, C4, and C7 planes, respectively). HP-CT = high pitch cervical CT, SD-CT = standard cervical CT, SNR = signal-to-noise ratio.

The SNR of the dural sac, paraspinal muscles, and trabecular bone was not statistically different at the upper (C2: 8.49 ± 2.78 vs 8.05 ± 2.25, 11.68 ± 3.94 vs 11.58 ± 3.28, and 18.88 ± 3.98 vs 19.77 ± 5.28; all, *P* > .05) and middle (C4: 9.75 ± 2.71 vs 8.44 ± 3.15, 13.28 ± 3.72 vs 13.12 ± 4.69, and 24.78 ± 7.10 vs 28.25 ± 9.42; all, *P* > .05) neck in the HP-CT and SD-CT, whereas that of the dural sac, paraspinal muscles, and trabecular bone at the lower (C7: 4.64 ± 2.15 vs 6.50 ± 3.63, 8.23 ± 2.84 vs 12.04 ± 3.91, and 14.68 ± 6.32 vs 20.21 ± 8.32; all, *P* < .05) neck in the HP-CT was lower when compared to that in the SD-CT, and the difference was statistically significant (Fig. 5).

**Figure. 6:**
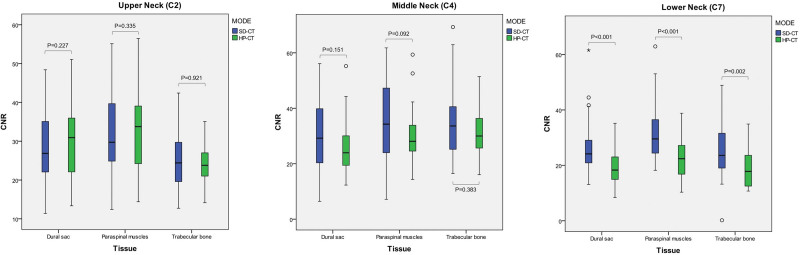
Comparison of CNR of three tissues at the upper, middle, and lower neck (corresponding to the C2, C4, and C7 planes, respectively). CNR = contrast noise ratio, HP-CT = high pitch cervical CT, SD-CT = standard cervical CT.

The CNR of the dural sac, paraspinal muscles, and trabecular bone was not statistically different at the upper (C2: 29.68 ± 9.94 vs 27.86 ± 8.38, 32.88 ± 11.05 vs 31.38 ± 9.54, and 24.28 ± 4.89 vs 24.42 ± 6.07; all, *P* > .05) and middle (C4: 26.77 ± 9.47 vs 30.51 ± 12.80, 30.30 ± 10.53 vs 35.20 ± 14.38, and 31.32 ± 8.54 vs 34.37 ± 11.43; all, *P* > .05) neck in the HP-CT and SD-CT, while that of the dural sac, paraspinal muscles, and trabecular bone at the lower (C7: 19.29 ± 7.19 vs 25.82 ± 8.39, 22.87 ± 7.92 vs 31.36 ± 9.31, and 19.15 ± 6.98 vs 25.51 ± 9.73; all, *P* < .05) neck in the HP-CT was lower when compared with that in the SD-CT, and the difference was statistically significant (Fig. 6).

In terms of subjective image quality, there was no significant difference in image quality subjective scores (both in bone window images and soft-tissue images) at the upper (C2), middle (C4), and lower (C7) neck between HP-CT and SD-CT (all, *P* > .05). The image quality of the SD-CT at the middle neck (C4) was featured with a higher subjective score both in the soft-tissue window images and bone window images, and it was better than that at the upper (C2)/lower (C7) neck. However, the image quality of the HP-CT at the lower neck (C7) got a lower subjective score, which was not as good as that of the upper (C2)/middle (C4) neck (Tables 2 and 3).

**Table 2:** Comparison of the subjective score of image quality at upper/middle/lower (correspondingly C2/C4/C7) neck and in total between SD-CT and HP-CT (soft-tissue window CT images).

Model	C2	C4	C7	Total
SD-CT (n = 66)*	4.44 (4.32–4.56)	4.86 (4.80–4.91)†	4.41 (4.29–4.53)	4.57 (4.51–4.63)
HP-CT (n = 29)*	4.35 (4.15–4.54)	4.83 (4.72–4.93)†	4.29 (4.15–4.43)	4.49 (4.39–4.59)
Z	−0.926	−0.317	−1.542	−1.520
*P* value	.355	.752	.123	.129

HP-CT = high pitch cervical CT, SD-CT = standard cervical CT.

* Data were given as mean (95%CI).

† The subjective score obtained on C4 is higher than that of C2 and C7 both in SD-CT and HP-CT, all *P* values < .05.

**Table 3:** Comparison of the subjective score of image quality at upper/middle/lower (correspondingly C2/C4/C7) neck and in total between SD-CT and HP-CT (bone window CT images).

Model	C2	C4	C7	Total
SD-CT (n = 66)*	4.20 (4.00–4.39)	4.64 (4.49–4.78)†	4.05 (3.84–4.25)	4.29 (4.18–4.40)
HP-CT (n = 29)*	4.40 (4.08–4.71)	4.59 (4.37–4.80)	3.76 (3.47–4.05)‡	4.25 (4.08–4.42)
Z	−1.256	−0.602	−1.811	−0.456
*P* value	.209	.547	.070	.649

HP-CT = high pitch cervical CT, SD-CT = standard cervical CT.

* Data were given as mean (95%CI).

† The subjective score obtained on C4 is higher than that of C2 and C7 in SD-CT, all *P* values < .05.

‡ The subjective score obtained on C7 is lower than that of C2 and C4 in HP-CT, all *P* values < .05.

### 3.3. Radiation dose

The mAs, CTDIvol, DLP, and effective dose in the HP-CT were all lower than those in the SD-CT, and the difference was statistically significant (all, *P* < .05; Table 4).

**Table 4:** Comparison of radiation dose in SD-CT and HP-CT.

Parameter	SD-CT (n = 66)	HP-CT (n = 29)	*P* value
mAs	196.86 ± 62.41	165.97 ± 29.35	.001
CTDIvol (mGy)	12.51 (10.07, 15.52)	10.69 (9.71, 11.35)	.002
DLP (mGy·cm)	238.80 (189.70, 291.63)	214.50 (195.70, 227.10)	.025
ED (mSv)	1.41(1.12, 1.72)	1.27 (1.15, 1.34)	.025

CTDIvol = volume CT dose index, DLP = dose–length product, ED = effective dose, HP-CT = high pitch cervical CT, SD-CT = standard cervical CT.

## 4. Discussion

Approximately 65% of spinal cord injuries occur in the cervical spine in the emergency procedure.^[15]^ Delayed treatment can lead to partial or complete paralysis in up to 29% of patients, and the probability of CSI in uncooperative patients is about three times that in alert patients.^[2, 16]^ In general, the clearance of CSI in moderate-high risk cervical spine trauma usually relies on the imaging evaluation, including the plain film, CT and MRI. Among them, the plain film has once played an important role in screening patients with low-medium risk CSI, but its effectiveness remains controversial at present.^[9, 17, 18]^ MRI possesses high sensitivity in detecting the injury in soft tissues and the spinal cord.^[10]^ However, it is often not the first option when considering contraindications and timeliness.^[18]^ As for cervical CT, it is effective in reducing the patient’s residence time in the emergency room and waiting time for treatment, due to its non-invasiveness, high accuracy, and wide range of applications.^[9,19]^ Therefore, cervical CT has been widely used in trauma patients for injury assessment, clinical decision-making, and efficacy evaluation.^[20]^

However, it remains challenging to successfully obtain CT images that meet the diagnostic requirements for the clearance of CSI in uncooperative patients with acute cervical spine trauma. Furthermore, the investigators could not determine the time-point of unpredictable movement caused by those uncontrollable factors during the cervical CT scan in this population. The cervical CT scan is a continuous process (approximately 6 s for SD-CT). In theory, movements occurring at any point during this process can lead to failure in cervical CT, and a repeated scan usually should be performed. What intrigued the investigators was that the success of the subsequent scan could not be guaranteed, and repeated CT scans cannot be indefinitely carried out. For example, the scan limitation in our institution is three times. Repeated CT scans of the spine due to various reasons are not uncommon.^[8, 21]^

The scan time for cervical CT is of vital importance. The pitch of the standard cervical CT ranges from 0.53 to 1.00, with a scan time of approximately 5 to 8 seconds.^[22-24]^ The longer the scanning time, the higher the probability of the movement artifact. An HP-CT protocol (with a pitch/scan time of 1.5/approximately 2 s, respectively) was introduced into uncooperative patients with cervical spine trauma, which could reduce the probability of movement during the scan, with a 73% scan time reduction, thus improving the success rate for one scan. In our study, a success rate of 83% in one CT scan was achieved using HP-CT. Besides, three patients were excluded because the first two CT scans were unsuccessful. However, it is still theoretically possible to obtain artifact-free images on a third scan. Since the movement artifacts appeared at different planes of the cervical spine in the previous two scans, the combination of these images could still meet the needs of diagnosis. To some extent, careful screening of “imperfect” images sufficient to answer clinical concerns may reduce repeated scans.

Regarding a previous study,^[5]^ the SNR/CNR of three representative tissues related to the cervical spine (namely, the bone structure-trabecular bone, supporting epidural soft tissue-paravertebral muscles, and tissues in the vertebrae tube-dural sac) was adopted to evaluate the image quality of the cervical CT, while that of these three tissues in the upper and middle neck performed similarly well, using the HP-CT and SD-CT. On one hand, it may benefit from the application of CT iterative reconstruction technology,^[25]^ while on the other hand, it may be correlated to a few overlapping tissues in the upper and middle neck.^[26]^ With a relatively higher CT value, the SNR for the cervical trabecular bone was higher than that for the other two tissues at the same plane, which was the primary concern of the investigators for screening fractures. Nevertheless, for the increments of the paraspinal muscles and the dural sac (the difference between itself and the background in the CT value), they were greater than those of the trabecular bone. The CNR of these three tissues reached a relative balance in the upper and middle neck, whereas the SNR/CNR of these three tissues at the lower neck was not as good as that at the upper/middle neck, both in the HP-CT and SD-CT, which may be related to increasing in overlapping tissues on the corresponding plane.^[26]^ Beyond that, the SNR/CNR at the lower neck in the HP-CT was not as good as that in the SD-CT. In addition to the overlapping tissues, this may also be correlated to the insufficient quantity of X-ray photons^[27]^ and older age^[28]^ in the HP-CT.

There was no significant difference in the subjective score of the image quality at the upper/middle or lower neck between the HP-CT and SD-CT groups, although poor performance still existed at the lower neck, which is consistent with the report of T.H. Mulkens et al.^[22]^ Similar to the results for the objective evaluation of image quality at the upper and middle neck, the subjective scores for image quality at the upper and middle neck were superior to that at the lower neck, using both the HP-CT and SD-CT. The reasons were likely to be similar to the causes of the difference in the objective image quality at the lower neck, including overlapping tissues^[26]^ and insufficient quantity of X-ray photons.^[27]^ In the opinion of the investigators, obtaining high-quality images at the lower neck by pulling down the shoulders of patients with acute trauma would be inappropriate, since secondary adverse events may occur. Meanwhile, the CT post-processing reconstructed images (such as MPR and VR) play an important role in the clearance of the CSI. In this case, the insufficiency of the diagnosis is made up for, relying on the axial cervical CT images. Hence, it has become an indispensable part of the diagnosis made by cervical CT.^[29, 30]^

Another practicality of the application of HP-CT is the radiation dose. It has been proved that the high-pitch mode reduces the radiation dose while maintaining high-quality images in the chest CT.^[31]^ In recent years, the application of advanced imaging, including CT, has increased to a great extent, which may be conducive to reducing the hospitalization rate in the emergency department.^[32]^ However, excessive radiological examinations on cervical spines would undoubtedly increase the risk of radiation exposure, thereby dramatically raising the patient’s risk of thyroid cancer.^[33, 34]^

According to the survey conducted in the UK in 2018,^[35]^ the average radiation dose was CTDIvol of 20 mGy/DLP of 440 mGy·cm (the third quartile, as shown below) when standard cervical CT (tube voltage 120 kV, automatic exposure control) was mostly adopted, and it was higher than the national diagnostic reference level in 2011 (correspondingly, CTDIvol of 15 mGy/DLP of 324 mGy·cm). In the present study, the radiation dose in both HP-CT and SD-CT groups was lower than that reported by J.R. Holroyd et al.^[35]^ The radiation dose in the SD-CT (correspondingly, CTDIvol of 16 mGy/DLP of 292 mGy·cm) was close to the national diagnostic reference level in 2011, while that in the HP-CT was lower when the same or sub-optimal images were obtained. In previous studies, the repeated scan caused by various reasons and the deviation of protocol settings in cervical CT may have resulted in a higher radiation dose. Considering that, a multi-win situation may be achieved among patients, physicians, and managers through the standardized set of the CT scan protocol, the refinement of the CT scan mode, and the top-level design, thus strengthening the sharing of image resources in different trauma centers/hospitals.

There were some limitations in this study. Although our conclusion was based on comparisons with SD-CT in terms of subjective image quality, objective image quality, and radiation doses, subsequent studies may require experimental model validation of positive samples in both scanning modalities. A statistically significant difference existed in the age of the groups in this study, which may have an impact on the results of the image quality evaluations. Hence, it may indicate that age is associated with tolerance to cervical spine trauma. The investigators did not consider the factors that may affect the measurement of the CT value, such as foreign bodies outside of cervical spines (including dentures and ear studs), diseases that can cause abnormal bone metabolism, and spinal cord or soft tissue diseases. The sample size is limited in the present study, especially for HP-CT. Considering that, studies with a larger population would be conducted for confirmation of our results.

## 5. Conclusions

With a significant reduction of both scan time and the radiation dose and similar image quality, it is feasible for HP-CT to be applied to uncooperative patients with acute cervical spine trauma.

## Acknowledgments

We would like to thank Huan Zhang (MD, from ruijin Hospital, Shanghai Jiaotong University School of Medicine, China) for her help in thoughtful suggestions for this paper.

## Author contributions

**Conception and design:** Jianchun Tu.

**Data curation:** Juntao Cao, Na Xie.

**Formal analysis:** Na Xie, Pingkang Qian.

**Investigation:** Juntao Cao, Na Xie, Pingkang Qian, Ming Hu.

**Methodology:** Juntao Cao, Na Xie.

**Resources:** Pingkang Qian, Ming Hu.

**Software:** Pingkang Qian, Ming Hu.

**Writing – original draft:** Juntao Cao, Na Xie.
